# Segmentation and counting of wheat spike grains based on deep learning and textural feature

**DOI:** 10.1186/s13007-023-01062-6

**Published:** 2023-08-01

**Authors:** Xin Xu, Qing Geng, Feng Gao, Du Xiong, Hongbo Qiao, Xinming Ma

**Affiliations:** 1 College of Information and Management Science, Henan Agricultural University, Zhengzhou, 450002 China; 2 Agricultural College, Henan Agricultural University, Zhengzhou, 450002 China

**Keywords:** Wheat ear, Grain number, Image processing, HRNet, Deep learning, Image segmentation

## Abstract

**Background:**

Grain count is crucial to wheat yield composition and estimating yield parameters. However, traditional manual counting methods are time-consuming and labor-intensive. This study developed an advanced deep learning technique for the segmentation counting model of wheat grains. This model has been rigorously tested on three distinct wheat varieties: ‘Bainong 307’, ‘Xinmai 26’, and ‘Jimai 336’, and it has achieved unprecedented predictive counting accuracy.

**Method:**

The images of wheat ears were taken with a smartphone at the late stage of wheat grain filling. We used image processing technology to preprocess and normalize the images to 480*480 pixels. A CBAM-HRNet wheat grain segmentation counting deep learning model based on the Convolutional Block Attention Module (CBAM) was constructed by combining deep learning, migration learning, and attention mechanism. Image processing algorithms and wheat grain texture features were used to build a grain counting and predictive counting model for wheat grains.

**Results:**

The CBAM-HRNet model using the CBAM was the best for wheat grain segmentation. Its segmentation accuracy of 92.04%, the mean Intersection over Union (mIoU) of 85.21%, the category mean pixel accuracy (mPA) of 91.16%, and the recall rate of 91.16% demonstrate superior robustness compared to other models such as HRNet, PSPNet, DeeplabV3+ , and U-Net. Method I for spike count, which calculates twice the number of grains on one side of the spike to determine the total number of grains, demonstrates a coefficient of determination R^2^ of 0.85, a mean absolute error (MAE) of 1.53, and a mean relative error (MRE) of 2.91. In contrast, Method II for spike count involves summing the number of grains on both sides to determine the total number of grains, demonstrating a coefficient of determination R^2^ of 0.92, an MAE) of 1.15, and an MRE) of 2.09%.

**Conclusions:**

Image segmentation algorithm of the CBAM-HRNet wheat spike grain is a powerful solution that uses the CBAM to segment wheat spike grains and obtain richer semantic information. This model can effectively address the challenges of small target image segmentation and under-fitting problems in training. Additionally, the spike grain counting model can quickly and accurately predict the grain count of wheat, providing algorithmic support for efficient and intelligent wheat yield estimation.

## Introduction

Traditional wheat yield estimation is obtained by manually investigating the number of spikes per unit area and the number of grains per spike and multiplying them with the conventional 1000-grain weight before wheat harvest. However, this method is time-consuming, inefficient, and prone to human error [1–3]. Image processing, machine learning, and computer vision can perform wheat yield estimation quickly and accurately. These technologies offer technical support and a solid foundation for obtaining phenotypic information about wheat plants [4], which can significantly enhance the timeliness and accuracy of wheat yield estimation.

Crop yield estimation is a critical scientific issue, with spike grain number being one of the most key parameters. Zhao et al. [5] have made a significant breakthrough in this field using a measuring method that better explains the correlation between the structural image features of rice spikes and the number of grains, providing a basis for estimating the number of grains of rice spikes. Wang et al. [6] combined phenotypic analysis, image processing, and deep learning to develop an intelligent phenotypic analysis algorithm that examined the number of grains per spike and various spike grain-related traits from rice spike images without threshing. Du et al. [7] have demonstrated the feasibility of studying spike grain number through structural features of the wheat spikelets, indirectly measuring it through the relationship between the number of pixels of wheat spikelet area and spike grain number. These studies have brought us closer to more accurate crop yield estimation.

Image processing and machine learning development has provided an important monitoring tool for segmenting and recognizing wheat and spike grain counts [8]. Although image processing techniques are widely used to identify the number of wheat ears and grains, there are still efficiency and practical application issues due to the extraction of texture, color, and morphological features. Du et al. [7] combined agronomic knowledge to segment wheat spikelets using fitted parabolas and counted the number of wheat spikelets and grains to obtain high accuracy of 97%. Fernandez-Gallego et al. [9] used the local maximum peak method to calculate the number of wheat spikes on RGB color images of large fields with a more than 90% success rate. However, current image processing techniques require a large amount of manual image feature extraction, which places high demands on the environment and technology. Machine learning has shown significant advantages in image segmentation and recognition. Liu et al. [10] proposed an algorithm for counting wheat ears based on K-means clustering of color features, with a recognition accuracy of 94%. Xu et al. [11] automatically extracted the contour features of wheat ears based on the K-means clustering algorithm and later built a Convolutional Neural Network (CNN) model to improve the accuracy of wheat ears recognition to 98.3%. Nevertheless, traditional image processing techniques and machine learning methods still face challenges, such as long recognition segmentation time, low efficiency, and poor complex image recognition segmentation effect [5, 12].

Modern methods of image analysis based on deep learning can achieve end-to-end detection of features in different domains, scenes, and scales. This method also has good feature extraction and generalization capabilities, widely used to identify the number of wheat ears and spikelets. Wei et al. [13] constructed a Faster R-CNN network model through the TensorFlow framework and used a counting model for migration learning techniques. By optimizing wheat seed detection, these authors achieved an error rate of less than 3% for the model. Hu et al. [14] proposed a generative adversarial network based on an attention mechanism to count the number of wheat ears and spikelets, achieving 84.9% of the F1 value for identifying wheat ears and segmenting spikelets. Dandrifosse et al. [15] used wheat images at the filling stage as the research object. They combined deep learning methods with wheat RGB images to achieve wheat ear counting in the field. The average F1 values for wheat ears detection and segmentation were 0.93 and 0.86, respectively. Zhao et al. [16] proposed an improved YOLOv5 method for accurately detecting wheat ears in UAV images, achieving 94.1% average accuracy (AP) of wheat ears detection. Although deep learning techniques have incomparable advantages in extracting wheat phenotypic information and achieving higher accuracy in image segmentation and recognition [17], acquiring images of wheat ears grain necessitate professional equipment such as CMOS cameras, which can be challenging to operate in complex production [18]. Furthermore, dense small targets pose challenging tasks for image recognition and segmentation because the adhesion between targets will likely occur, making accuracy improvement challenging [4, 14].

The primary objective of this study is to enhance the precision and effectiveness of wheat spikelet identification, segmentation, and counting, designing three varieties of field experiments: ‘Bainong 307’, ‘Xinmai 26’, and ‘Jimai 336’, using mobile terminals to capture images of wheat spikelets. This study also constructs a deep learning segmentation model for wheat spikelet grain count, which is further processed by applying image processing techniques and combining the segmentation results of spike grains with the texture features of wheat spikelets. This process builds a spike grain count model that accurately predicts the grain count of wheat spikelets. The main goal of this approach is to obtain fast and efficient segmentation results and achieve a precise wheat yield estimation.

## Materials and methods

### Experimental design

The experiment was conducted at the Yuanyang Science and Education Park of Henan Agricultural University (35°6′46 ʺN, 113°56′51 ʺE). The main wheat varieties selected were ‘Bainong 307’, ‘Xinmai 26’, and ‘Jimai 336’. A split-zone design was used, with nitrogen application as the main zone and varieties as secondary zones. Nitrogen fertilizer treatments included N15 (225 kg/hm^2^) and N19 (285 kg/hm^2^). The trials were randomly arranged between varieties and replicated three times, with an area of 49.33 m^2^ per plot. The seeding rate of wheat was the best sowing rate proposed locally, i.e., 12.5 kg/acres for ‘Bainong 307’, 12.5 kg/acres for ‘Xinmai 26’, and 9.5 kg/acres for ‘Jimai 336’, with a sowing date of 23 October 2021 and a row spacing of 20 cm. The trial was fertilized with urea (46%) for nitrogen fertilizer, Calcium superphosphate (12%) for phosphate fertilizer, and Potassium chloride (60%) for potash, with a 6:4 ratio of base to chase nitrogen fertilizer. The chase fertilizer was applied at nodulation. The phosphate and potash fertilizers were applied as base fertilizers. Other field management was similar to those general high-yielding fields.

### Data acquisition

**Fig. 1:**
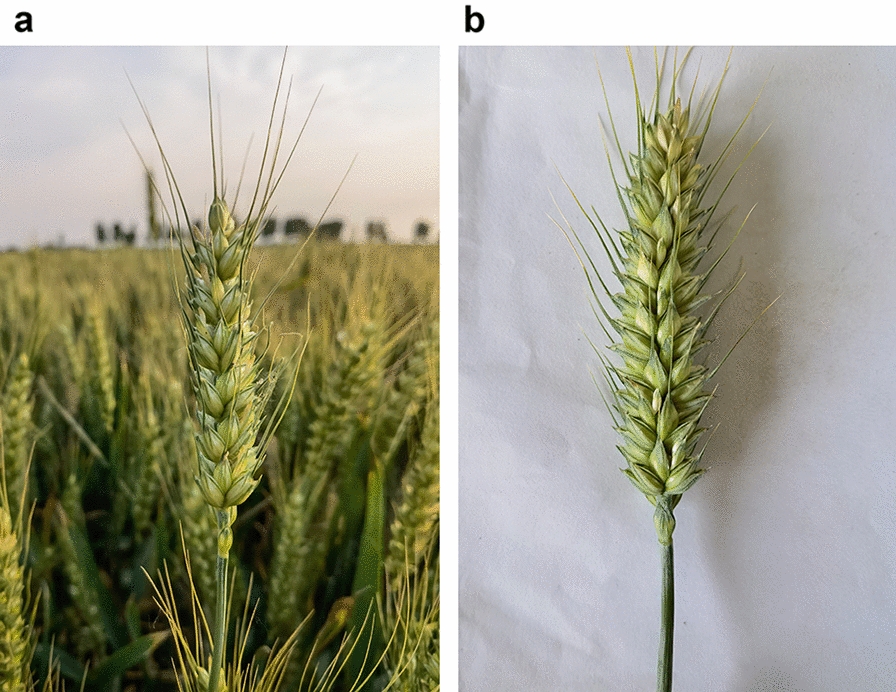
Original wheat ear image. **a** images obtained by in situ sampling **b** images obtained by off-body sampling. the distance is adjusted to provide a clear image of the whole wheat ear while shooting

The experiment was performed to sample wheat during the filling stage. We used two image acquisition devices: HUAWEI Mate 40 Pro (50-megapixel primary camera and 20-megapixel secondary camera) and Realme Q3 with 48-megapixel primary camera and 8-megapixel secondary camera. The specific shooting time was conducted from 9:00 a.m. to 4:00 p.m. The experiment used two types of image acquisition: off-body sampling in the laboratory environment and in situ sampling in the field environment to increase the model’s complexity, diversity, and generalization level [4]. Each wheat ear’s spikelets and seeds were counted manually after image acquisition. To acquire the image, we held the mobile device parallel to the wheat ears and adjusted the vertical height until the ears were entirely in view, showing a clear image of the ears. Refer to Fig. 1 for the original image of the ears of wheat obtained according to this method.

We selected 30–40 plants for each wheat variety with each nitrogen fertilizer treatment and different shooting backgrounds, resulting in 660 original wheat ears images. Table 1 shows the wheat ears dataset.

**Table 1:** Wheat ear dataset information

Wheat varieties	Nitrogen fertilizer treatment	Shoot background	Shoot data	Weather	Resolution/mm	Image size	Shoot device	Focal length/mm	Number of images
Bainong 307	N15	Wheatfield	15/05/2022	Sunny	0.26–0.56	3072 4096	HUAWEI Mate 40 Pro	7	10
	N15	Wheatfield	15/05/2022	Sunny	0.35–0.54	3000 4000	Realme Q3	5	10
	N15	White paper	08/06/2022	Cloudy	0.26–0.56	3680 5408	HUAWEI Mate 40 Pro	4	90
	N19	Wheatfield	15/05/2022	Sunny	0.26–0.56	3072 4096	HUAWEI Mate 40 Pro	7	10
	N19	Wheatfield	15/05/2022	Sunny	0.35–0.54	3000 4000	Realme Q3	5	10
	N19	White paper	08/06/2022	Cloudy	0.26–0.56	3680 5408	HUAWEI Mate 40 Pro	4	90
Xinmai 26	N15	Wheatfield	15/05/2022	Sunny	0.26–0.56	3072 4096	HUAWEI Mate 40 Pro	7	10
	N15	Wheatfield	15/05/2022	Sunny	0.35–0.54	3000 4000	Realme Q3	5	10
	N15	White paper	08/06/2022	Cloudy	0.26–0.56	3680 5408	HUAWEI Mate 40 Pro	4	90
	N19	Wheatfield	15/05/2022	Sunny	0.26–0.56	3072 4096	HUAWEI Mate 40 Pro	7	10
	N19	Wheatfield	15/05/2022	Sunny	0.35–0.54	3000 4000	Realme Q3	5	10
	N19	White paper	08/06/2022	Cloudy	0.26–0.56	3680 5408	HUAWEI Mate 40 Pro	4	90
Jimai 336	N15	Wheatfield	15/05/2022	Sunny	0.26–0.56	3072 4096	HUAWEI Mate 40 Pro	7	10
	N15	Wheatfield	15/05/2022	Sunny	0.35–0.54	3000 4000	Realme Q3	5	10
	N15	White paper	08/06/2022	Cloudy	0.26–0.56	3680 5408	HUAWEI Mate 40 Pro	4	90
	N19	Wheatfield	15/05/2022	Sunny	0.26–0.56	3072 4096	HUAWEI Mate 40 Pro	7	10
	N19	Wheatfield	15/05/2022	Sunny	0.35–0.54	3000 4000	Realme Q3	5	10
	N19	White paper	08/06/2022	Cloudy	0.26–0.56	3680 5408	HUAWEI Mate 40 Pro	4	90

Each side of the wheat ears was photographed to expand the dataset

In addition to the data listed in Table 1, five additional wheat varieties were selected for image acquisition in May 2023 at the Yuanyang Science and Education Park of Henan Agricultural University. The five wheat varieties selected were ‘Bainong 4199’, ‘Kexing 3302’, ‘Yangmai 15’, ‘Yunong 904’, and ‘Zhengmai 136’. The image acquisition devices we used was HUAWEI Mate 40 Pro and a total of 50 images were taken to verify the generalization ability of the model.

### Technical route

**Fig. 2:**
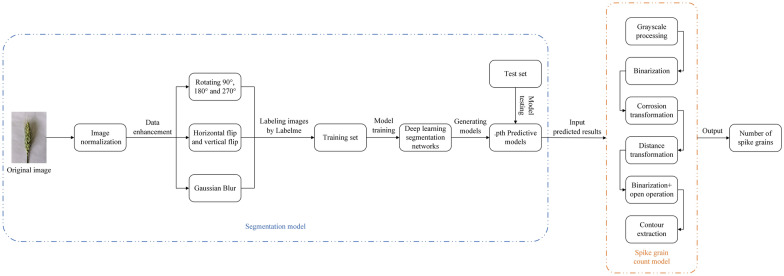
Original wheat ear image. the spike grain count model and data processing are implemented through the image processing library in python. labelme achieves data annotation and label format conversion

Figure 2 depicts the proposed technical route. The first crucial step is preprocessing the wheat spikelet images and forming a dataset. Then, a deep learning segmentation network is used to segment the image of wheat spike grain, followed by training the prediction model. Subsequently, the prediction model is used to test the test set. Then, image processing techniques are employed to construct a spike grain count model and obtain accurate prediction and counting of wheat spike grains.

### Data processing

#### Data normalization

Data normalization is a crucial step in preparing images for model training, employed to convert all images into a uniform size to make the model’s training process more efficient. The original wheat images are too large and demanding for the equipment. Thus, normalizing the original images is crucial to balance the device’s computing power with the number and quality of images [16]. By normalizing the image size in the dataset to 480*480 before model training, we can reduce the number of model operations and the risk of overfitting.

#### Data enhancement

The number of images can significantly impact the test accuracy and results when training a machine learning model. Insufficient images can decrease test accuracy, making data enhancement an essential technique to address the issues of insufficient images, using images from the original dataset for expansion [14]. Random cropping may remove feature information from the wheat images and enhance the dataset by rotating the images by 90°, 180°, and 270°, flipping them horizontally and vertically. Additionally, Gaussian blur was used to reduce image noise and detail by constantly resizing the Gaussian convolution kernel to find the most suitable Gaussian blur process. After comparing images at different scale sizes, the size of the Gaussian convolution kernel was set to 5*5. This process is the most effective way to enhance the image at different scale sizes.

#### Dataset construction

This study adopts a supervised learning model, entailing a deep learning model with manually annotated data samples to create a network model with specific generalization capabilities for computer vision tasks such as target classification, target detection, and image segmentation [19]. The Labelme image annotation tool manually marks and converts two types of segmentation objects, wheat spike grain and background, into masked images. These images, along with their mask images, formed the dataset required for the deep learning segmentation model. The number of images processed was evenly distributed for each of the three varieties in the dataset. The wheat ears dataset was divided into a training set and a test set in a ratio of 9:1 [11], with 594 images and 66 images in the test set. Finally, the test set was used as the validation set with 66 images.

### Model construction

Segmenting wheat spike grain is an incredibly challenging task, as the grains are located close to each other in terms of pixel points. This issue makes the images heavily sticky, which calls for high resolution and global information acquisition ability of deep learning models. This study constructed CBAM-HRNet based on CBAM, HRNet, PSPNet, DeeplabV3+ segmentation model, and U-Net for accurate segmentation and computational efficiency.

#### CBAM-HRNet

CBAM-HRNet is the ultimate solution for achieving strong semantic information and accurate positional information by parallelizing multiple resolution branches and constant information interaction between branches without losing much valid information during constant upsampling. After converting the spikelet images in the dataset into feature maps, different fusion outputs are obtained by the Stage structure, parallel convolutional branching, and multi-resolution fusion modules. For the semantic segmentation task, the representation branch is structured so that low-resolution features are up-sampled to increase the resolution, stacked, and fused to form a spike grain prediction map [20].

The CBAM is incorporated to achieve optimal results in the upsampling process of the representation branch. CBAM combines channel attention and spatial attention mechanisms, proving more efficient than using attention mechanisms as it focuses only on channels or space. The channel and spatial attention mechanisms process the input feature layer. The channel attention mechanism performs global average pooling and maximum global pooling on the individual feature layers of the input, which are then processed using a shared fully connected layer. After this, the Sigmoid activation function is used to obtain a weight (between 0 and 1) of each channel of the input feature layer. Then, this weight is multiplied by the original input feature layer to complete the process. The spatial attention mechanism takes the maximum and average values for the input feature layer on each channel of the feature point. Then, the results are stacked, and the number of channels is adjusted using a convolution with one channel at a time [21].

**Fig. 3:**
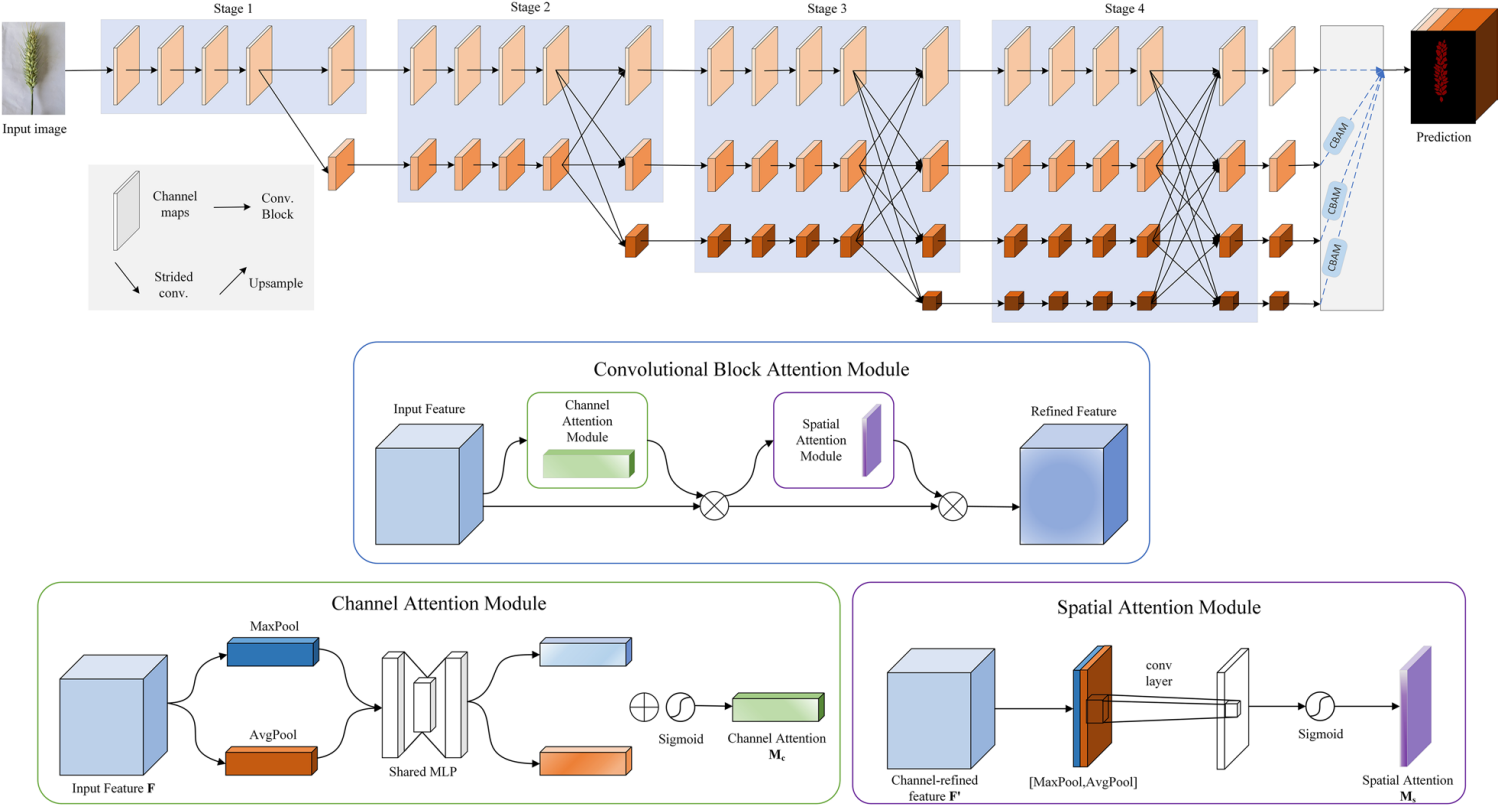
Network structure of wheat grain segmentation based on CBAM-HRNet. the convolutional block attention module is added to the upsampling process of the representation branch

CBAM-HRNet network architecture is the perfect fit for location-sensitive semantic segmentation tasks. Its ability to maintain high resolution from start to finish is unmatched. The interaction of information from different branches can supplement the information loss caused by the reduced number of channels. Additionally, the adaptive attention to the network offers significant advantages. Figure 3 presents the CBAM-HRNet network structure.

#### PSPNet

**Fig. 4:**
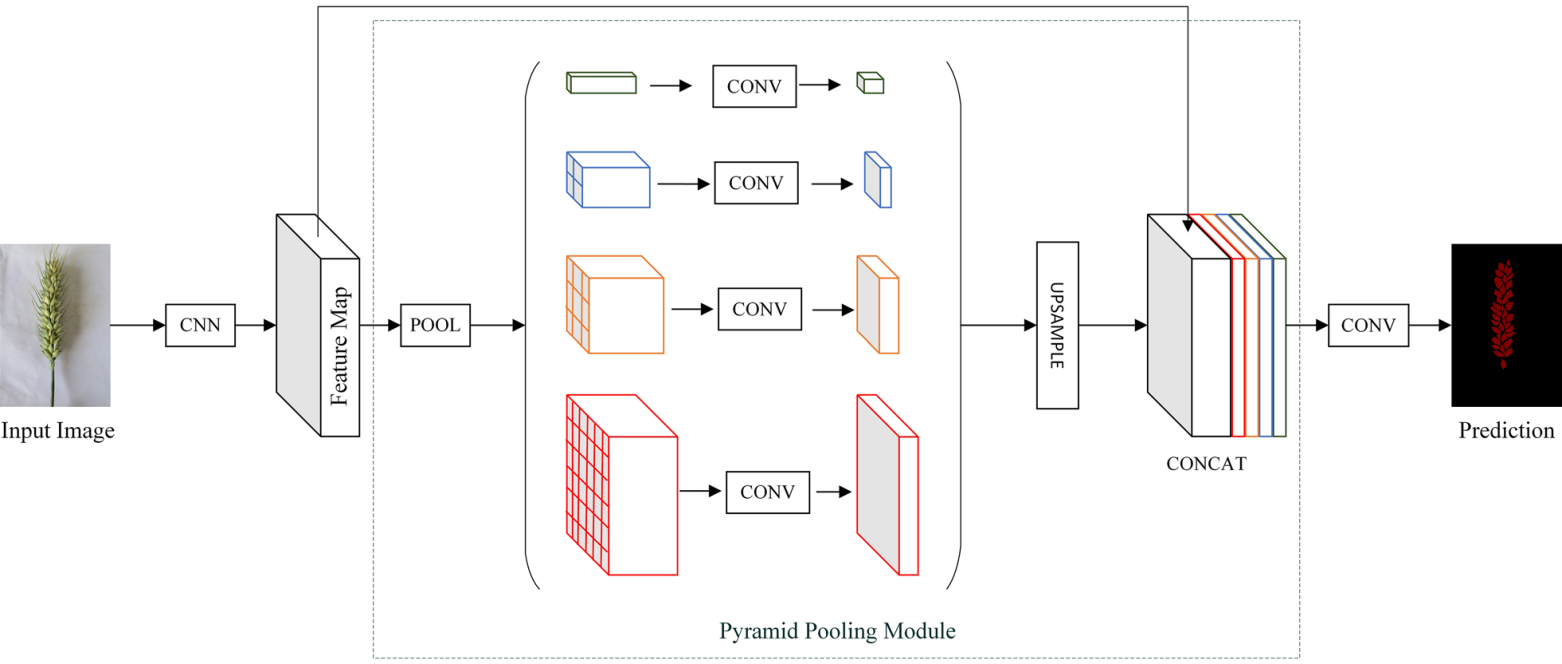
Network structure of wheat grain segmentation based on PSPNet

PSPNet is an enhanced version of a Fully Convolutional Network, using a ResNet network with added dilated convolution as the feature extraction network for the input wheat image. The extracted features are fed into the Pyramid Pooling Module to obtain pyramid features of different depths and reduce dimensionality. These pyramid features are then upsampled and merged to produce a final spike grain feature map [22]. Figure 4 depicts the network structure of PSPNet.

#### DeeplabV3+ segmentation model

**Fig. 5:**
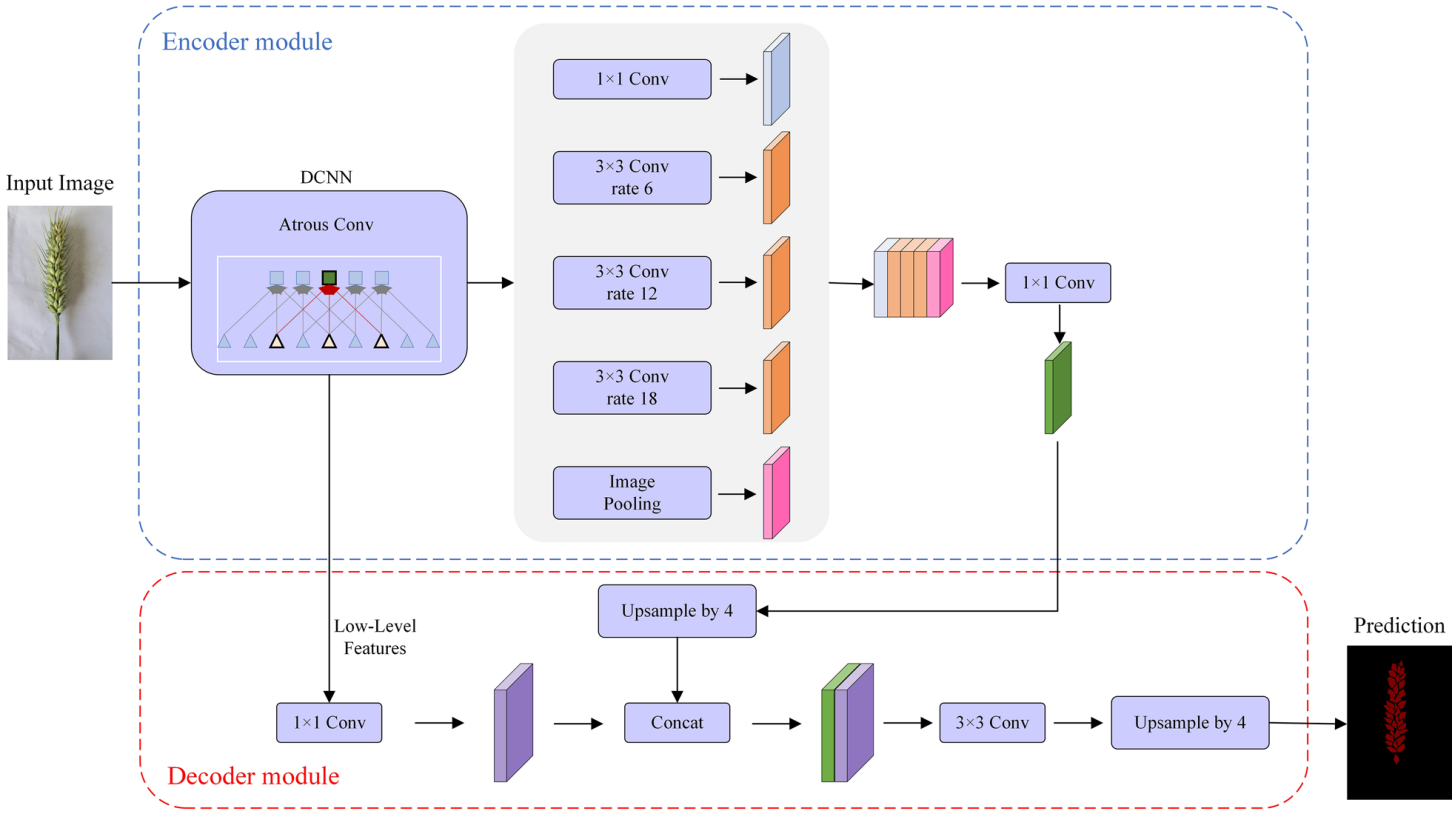
Network structure of wheat grain Segmentation based on DeeplabV3+ model

The DeeplabV3+ segmentation model is an encoder–decoder structure [23] that enables the most accurate wheat spikelet images. In the encoder part, the model uses Backbone and Atrous Spatial Pyramid Pooling to obtain five feature maps and fuse them, utilized as input to the decoder [24]. The decoder part involves channel downscaling, interpolation upsampling, and convolution block to generate a spike grain prediction map with the same resolution size as the original map [25]. The network structure of the DeeplabV3+ segmentation model is shown in Fig. 5.

#### U-Net

**Fig. 6:**
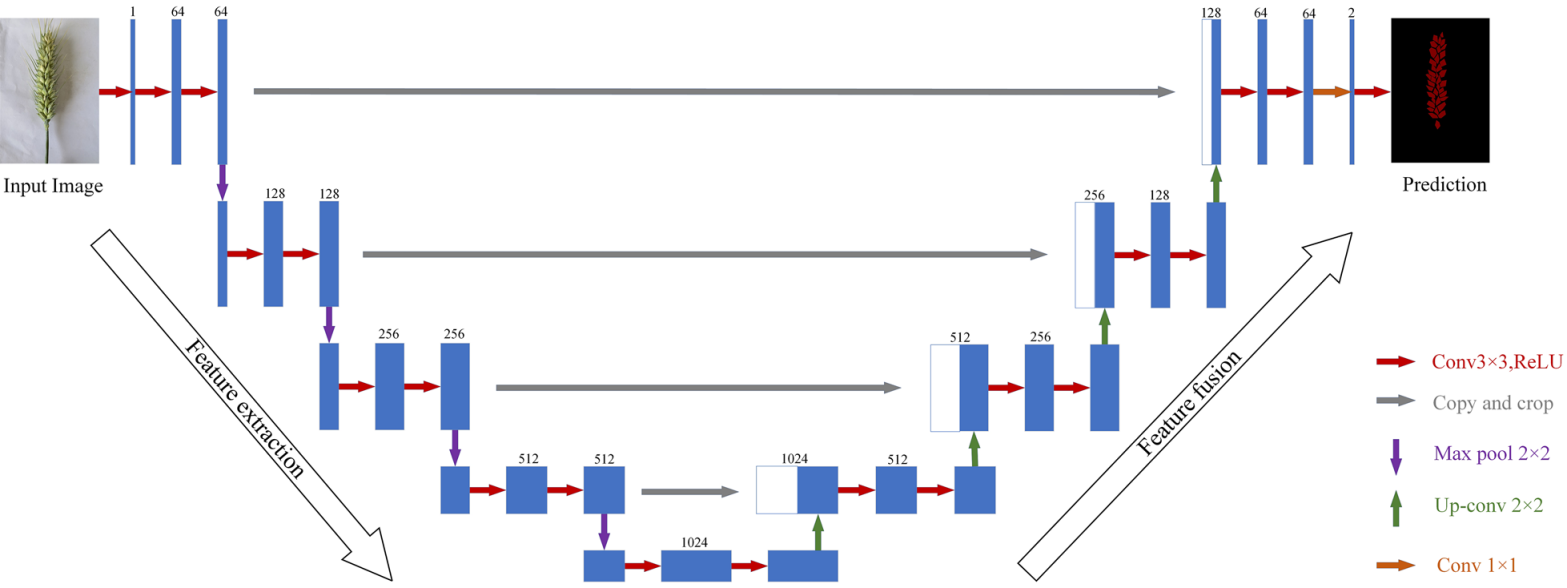
Network structure of wheat grain segmentation based on U-Net

U-Net is an exceptional deep learning network with an encoder–decoder architecture. The encoder extracts spike grain features from the wheat images in the dataset, using a convolution module and a pooling layer to obtain a high-level feature vector of the input image. This vector is then input into the decoder [26], which increases the feature image resolution through deconvolution and decodes using a decoding module. This softmax layer determines the probability that a pixel in the feature map belongs to the spike grain class, which determines the class of the pixel accordingly. The final result of the spike grain segmentation is generated [27]. Figure 6 provides details of the U-Net network structure.

### Spike grain count model

The wheat grains are generally present in pairs on both sides of the rachilla. Two methods are used to count the grains based on the geometric and textural characteristics of the wheat ears. Method I involves doubling the number of grains on one side of the ears to determine the total number of spike grains. Method II involves adding the grains on both sides of the total number of spike grains [5, 7, 12].

**Fig. 7:**
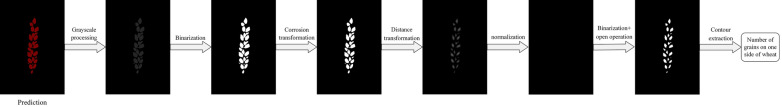
Spike grain count model. the model is mainly implemented with OpenCV and NumPy in python and requires the prediction results of the deep learning model as input

The distance between the mobile device and the spikelet was kept essentially the same during image acquisition, excluding the effect of different shooting distances on the wheat spike grains [5, 7]. After the deep learning segmentation model has predicted the selected samples of wheat ears from each variety, there will be instances of adhesion between the individual spike grains requiring image processing methods to eliminate the overlap and adhesion. The prediction results undergo grayscale processing with a color space conversion from RGB to GRAY via OpenCV and NumPy [28]. Then, a threshold of 120 is applied for binarization, with pixels greater than those threshold set to 255 (i.e., White) and those less than this threshold set to 0 (i.e., Black). The binarized image must undergo erosion transformation to eliminate noise and calculate the distance between the pixel point in the image and the nearest zero pixel point. The outline skeleton is obtained after the distance transformation, and the binarization effectively removes the overlapping part. Next, the dimensioned expression is converted into a dimensionless expression using normalization to become a scalar, and the grayscale value of the image is obtained between 0 and 1.0 after normalization. Subsequently, the grayscale image is processed into a binarized image through binarization and open operations. The original overlapping parts no longer overlap, and the shape is drawn according to the boundary points provided for contour extraction. The number of contours extracted is the number of wheat spike grains on one side [29]. The process of the spike grain count model is outlined in Fig. 7.

### Evaluation indicators

Several indicators are used to evaluate the segmentation model’s accuracy: Precision, Recall, Mean Pixel Accuracy (mPA), and Mean Intersection over Union (mIoU). The evaluation indicators are calculated from the parameters in the confusion matrix. In evaluating model accuracy, the confusion matrix is mainly used to compare the predicted and true values, which is used to compare the position of each true image element with the predicted image element [9]. Precision measures the proportion of the predicted values that are true. Recall is the proportion of true values that are predicted correctly. Intersection over Union is a standard metric used to evaluate the accuracy of semantic segmentation. However, mPA is the proportion of pixels per category that are correctly classified; mIoU is the average of all categories of IoU. The formulas are as follows:

1 $${\text{Precision}} = \frac{{{\text{TP}}}}{{{\text{TP}} + {\text{FP}}}}{.}$$

2 $${\text{Recall}} = \frac{{{\text{TP}}}}{{{\text{TP}} + {\text{FN}}}}{.}$$

3 $${\text{IoU}} = \frac{{{\text{TP}}}}{{{\text{TP}} + {\text{FP}} + {\text{FN}}}}.$$

4 $${\text{mPA = }}\frac{{{\text{sum}}\left( {{\text{P}}_{{\text{k}}} } \right)}}{{\text{k}}}.$$

5 $${\text{mIoU}} = \frac{1}{k + 1}\sum\limits_{k}^{i\, = \,0} {\frac{{{\text{TP}}}}{{\text{TP + FP + FN}}}} .$$

where *TP* is the number of positive samples predicted to be true by the segmentation model; *TN* is the number of negative samples predicted to be false by the segmentation model; *FP* is the number of negative samples predicted to be true by the segmentation model; *FN* is the number of positive samples predicted to be false by the segmentation model; *k* is the total number of categories; *P*_*k*_ is pixel accuracy per category.

The number of grains per sample was counted manually as the true value. The image segmentation algorithm obtained the number of grains per sample, and the spike-grain prediction model was used as the predicted value. To quantify the accuracy of the counting model, we used root mean square error (RMSE), mean absolute error (MAE), mean relative error (MRE), and coefficient of determination (R^2^) [30] as our metrics. These metrics can determine the accuracy of the segmentation and counting models when analyzing the number of grains per sample. We can improve the models by analyzing these metrics and optimizing their performance for better accuracy.

6 $$p_{i} = \frac{{\left| {x_{i} - y_{i} } \right|}}{{x_{i} }} \times 100\% {.}$$

7 $$R^{2} = 1 - \frac{{\sum\limits_{i = 1}^{n} {\left( {x_{i} - y_{i} } \right)^{2} } }}{{\sum\limits_{i = 1}^{n} {\left( {x_{i} - y_{i} } \right)^{2} } }}.$$

8 $$RMSE = \sqrt {\frac{1}{n}\sum\limits_{i = 1}^{n} {\left( {x_{i} - y_{i} } \right)^{2} } } .$$

9 $$MAE = \frac{1}{n}\sum\limits_{i = 1}^{n} {\left| {x_{i} - y_{i} } \right|} .$$

10 $$MRE = \frac{1}{n}\sum\limits_{i = 1}^{n} {p_{i} } \times 100\% .$$

where *P*_*i*_ is a relative error for a single sample; *x*_*i*_ is the true number of grains on the spike; *y*_*i*_ is the predicted number of grains on the spike; $\overline{x }$ is the average of the true values of the number of grains on the spike; *n* is the number of samples per variety.

## Performance analysis

For this study, we used a CPU with an Intel(R) Xeon(R) Silver 4114 CPU @ 2.20-GHz processor, 64 GB of memory, and a GPU provided by an NVIDIA Corporation GP104G with 16 GB of video memory. We ran all comparison algorithms in the same environment, which included the Ubuntu operating system and the PyTorch 1.12 deep learning framework compiled in Python 3.9.12.

Based on our analysis, the network is poorly trained when starting from 0, as the random weights impact the feature extraction [30]. A freeze-unfreeze mechanism was constructed based on transfer learning to address this issue. The pretraining weight of the model is shared across different datasets for the effectiveness of the features extracted. Without pretraining this weight, the backbone feature extraction part of the neural network may contain random weights leading to a poor network. The pretraining weight must be used in most cases. Otherwise, the weight in the backbone part may be too random for the feature extraction to be effective, making the network training results negative. In contrast, freezing up the training can significantly speed up the training efficiency and prevent the weight from being corrupted [31]. In the freezing phase, the model backbone is frozen; the feature extraction network remains unchanged, and the occupied video memory becomes small. However, only the network is fine-tuned to meet the training needs of different machine performances. In the unfrozen phase, the model backbone is no longer frozen; the feature extraction network is altered, The occupied video memory becomes larger, and all network parameters are changed.

The training parameters for the freeze phase are as follows: the current training generation of the model (Init_Epoch = 0), the number of iterations of the model freeze training (Freeze_Epoch = 50), and the batch size of the model freeze training (Freeze_batch_size = 16). The training parameters for the unfreezing phase are as follows: the total number of iterations of the model training (UnFreeze_Epoch = 300) and the batch size of the model after unfreezing (Unfreeze_batch_size = 8).

### Training results of the wheat spike grain segmentation model

**Fig. 8:**
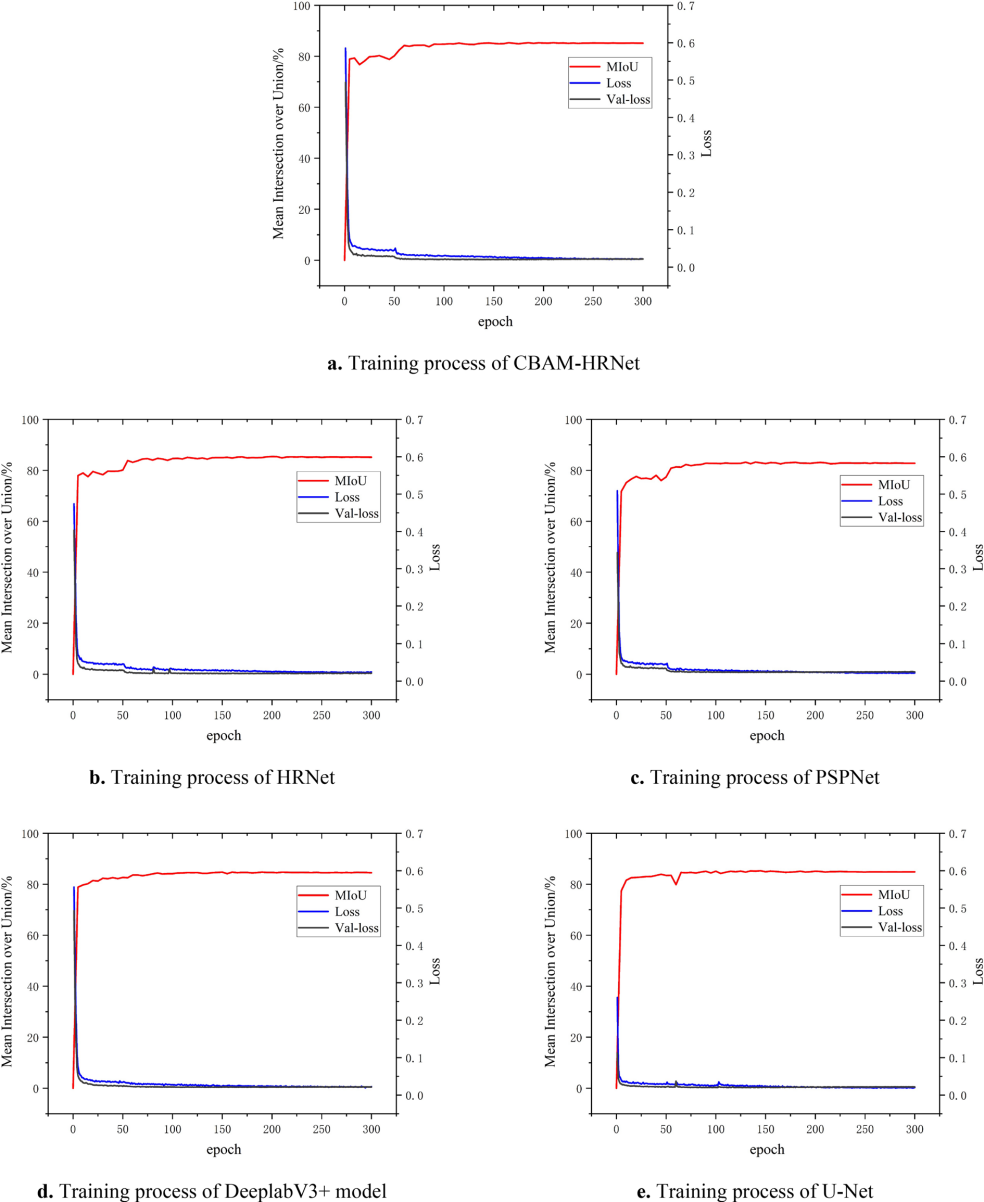
Performance curves of different models in the training process. The backbone and optimizers for these models are optimal, and the training process for these models is shown as examples

After thoroughly analyzing the wheat ears training set using the CBAM-HRNet, HRNet, PSPNet, DeeplabV3+ segmentation model, and U-Net, we compared the mIoU and loss values. It is evident from Fig. 8 that all five models steadily increased the mIoU values during the continuous iterations, which gradually converge steadily with the increase in the number of iterations. The mIoU value of the CBAM-HRNet based on the CBAM was stable at around 0.85, indicating a superior segmentation effect on the wheat ears dataset. Additionally, the loss value of the model in the training and validation sets decreased rapidly and gradually converged to around 0.021. The network converged quickly, with no sudden increase in the error, and the magnitude of the error change was very gentle. The difference in error between the two datasets is negligible, indicating that the model can find the appropriate gradient direction quickly and accurately during the gradient calculation, thereby offering stable performance and a good learning effect. The trend of loss values in the training and validation sets is the same, indicating that the model has good generalization ability.

### Performance comparison of wheat spike grain segmentation models

Different backbone networks, optimizers, and learning rates were used following various segmentation models to measure the effectiveness of the proposed models. Table 2 shows the results of our evaluation, using various indicators to compare the performance of the model training. The values were calculated as the means over the ten folds, providing a comprehensive overview of the model’s performance.

**Table 2:** Performance comparisons and evaluation of different segmentation models

Model	Backbone	Optimizer	Learning rate	Weight decay	Recall	Precision	Mean intersection over union	Mean pixel accuracy
CABM-HRNet	**hrnetv2_w32**	**Adam**	0.0005	0	**0.9116 ± 0.099**	**0.9204 ± 0.107**	**0.8521 ± 0.034**	**0.9116 ± 0.099**
	hrnetv2_w18	Adam	0.0005	0	0.9061 ± 0.097	0.9174 ± 0.138	0.8500 ± 0.027	0.9061 ± 0.097
	hrnetv2_w32	SGD	0.004	0.0001	0.8953 ± 0.099	0.9122 ± 0.123	0.8360 ± 0.023	0.8953 ± 0.099
	hrnetv2_w18	SGD	0.004	0.0001	0.8939 ± 0.100	0.9053 ± 0.168	0.8302 ± 0.029	0.8939 ± 0.100
HRNet	**hrnetv2_w32**	**Adam**	0.0005	0	**0.9100 ± 0.110**	**0.9189 ± 0.131**	**0.8510 ± 0.042**	**0.9100 ± 0.110**
	hrnetv2_w18	Adam	0.0005	0	0.9097 ± 0.125	0.9176 ± 0.142	0.8505 ± 0.040	0.9097 ± 0.125
	hrnetv2_w32	SGD	0.004	0.0001	0.8910 ± 0.126	0.9140 ± 0.130	0.8341 ± 0.043	0.8910 ± 0.126
	hrnetv2_w18	SGD	0.004	0.0001	0.8896 ± 0.175	0.9064 ± 0.139	0.8278 ± 0.056	0.8896 ± 0.175
PSPNet	MobileNetv2	Adam	0.0005	0	0.8995 ± 0.149	0.8883 ± 0.124	0.8221 ± 0.052	0.8995 ± 0.149
	**ResNet50**	**Adam**	0.0005	0	**0.9018 ± 0.230**	**0.8939 ± 0.119**	**0.8278 ± 0.052**	**0.9018 ± 0.230**
	MobileNetv2	SGD	0.01	0.0001	0.8566 ± 0.498	0.8554 ± 0.177	0.7718 ± 0.239	0.8566 ± 0.498
	ResNet50	SGD	0.01	0.0001	0.8777 ± 0.315	0.8900 ± 0.148	0.8082 ± 0.122	0.8777 ± 0.315
DeeplabV3 +	**MobileNetv2**	**Adam**	0.0005	0	**0.9101 ± 0.151**	**0.9118 ± 0.147**	**0.8468 ± 0.051**	**0.9101 ± 0.151**
	Xception	Adam	0.0005	0	0.9060 ± 0.135	0.9100 ± 0.143	0.8425 ± 0.038	0.9060 ± 0.135
	MobileNetv2	SGD	0.007	0.0001	0.8994 ± 0.265	0.8826 ± 0.148	0.8178 ± 0.064	0.8994 ± 0.265
	Xception	SGD	0.007	0.0001	0.8945 ± 0.265	0.8843 ± 0.109	0.8158 ± 0.055	0.8945 ± 0.265
U-Net	ResNet50	Adam	0.0001	0	0.9055 ± 0.103	0.9172 ± 0.145	0.8473 ± 0.035	0.9055 ± 0.103
	**VGG**	**Adam**	0.0001	0	**0.9045 ± 0.044**	**0.9198 ± 0.162**	**0.8484 ± 0.014**	**0.9045 ± 0.044**
	ResNet50	SGD	0.01	0.0001	0.8892 ± 0.303	0.8944 ± 0.142	0.8192 ± 0.084	0.8892 ± 0.303
	VGG	SGD	0.01	0.0001	0.8948 ± 0.084	0.9057 ± 0.137	0.8312 ± 0.020	0.8948 ± 0.084

Different backbone, optimizers, and learning rates were used according to different segmentation models. The evaluation indicators were measured on the test set with ten-fold cross-validation (mean ± standard deviation). The best results of each network are shown in bold

The results indicate that the CBAM-HRNet model with hrnetv2_w32 as the backbone network and Adam as the optimizer outperformed the other models in terms of segmentation accuracy, achieving a remarkable mIoU of 0.8521. The HRNet model with hrnetv2_w32 as the backbone network and Adam as the optimizer came second with mIoU = 0.851. The PSPNet model with MobileNetv2 as the backbone network and SGD as the optimizer had the lowest segmentation accuracy (mIoU = 0.7718). The CBAM-HRNet model demonstrates superior segmentation accuracy in almost all cases, proving that the CBAM generates more affluent spatial attention that complements the channel attention effectively. The overall CBAM model’s overhead is minimal in terms of both parameters and computation, as shown in Table 3, making CBAM-HRNet achieve better results than HRNet with slightly increased parameter and computational overhead. The five models trained using the Adam optimizer outperformed those using the SGD optimizer because Adam solved the problem of the SGD optimizer’s slow descent rate and could obtain local optimal solutions by combining first-order and second-order momentum with adaptive learning rates [9].

**Table 3:** The five networks that achieved the best results were compared regarding parameters and computational time over ten-fold cross-validation (mean ± standard deviation)

Model	Backbone	Optimizer	Number of parameters/million	Training time (s/epoch)	Segmentation time (ms/image)
CABM-HRNet	hrnetv2_w32	Adam	30.598	90.75 ± 0.825	12.75 ± 0.093
HRNet	hrnetv2_w32	Adam	29.547	91.02 ± 0.559	12.65 ± 0.052
PSPNet	ResNet50	Adam	2.377	108.56 ± 0.658	9.98 ± 0.076
DeeplabV3 +	MobileNetv2	Adam	5.818	89.29 ± 0.612	8.62 ± 0.105
U-Net	VGG	Adam	24.892	138.11 ± 1.744	9.59 ± 0.070

CBAM has a lower overhead and computational load

Based on the statistical analysis performed in Table 2, Paired t-tests were used to determine whether the mIoU of CBAM-HRNet and HRNet were significantly different. The null hypothesis is rejected if the p-Value < α < 0.5, indicating that the differences in the model results are so convincing at the 95% confidence level that they can be considered significant. The t-test on the mIou of CBAM-HRNet and HRNet yields the p-Value of 0.0497, indicating that the null hypothesis is rejected and the mIou is significantly different. Therefore, we can conclude that the improvement in CBAM-HRNet accuracy is not due to network chance but improve the segmentation accuracy of the model.

### Comparison of wheat spike grain segmentation effects

**Fig. 9:**
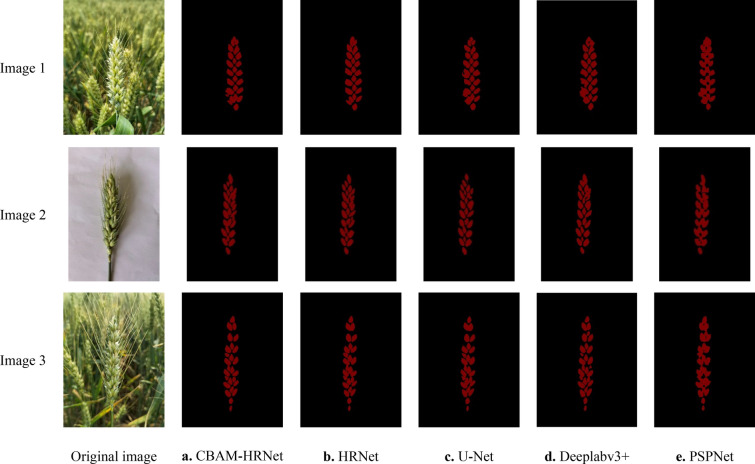
The segmentation effect of the different models on the test dataset. The backbone and optimizers for these models are optimal, and CBAM-HRNet has the best segmentation effect among them

Figure 9 shows the segmentation results, which compare and analyze the segmentation ability of the CBAM-HRNet based on CBAM, HRNet, U-Net, PSPNet, and DeeplabV3+ segmentation model based on the wheat spikelet test set. The results indicate that the segmentation effect of PSPNet and DeeplabV3+ models is unsatisfactory. Although they can distinguish between the wheat spike grains and the background, they are still stuck together. Divining spike grains and the pixel point values is challenging based on similarities in their grayscale features. The difference in grayscale value between the background and the wheat spikelets is quite significant, with the background being much darker than the wheat spikelets. Thus, it is easy to distinguish and separate the two based on this background [28, 29]. In contrast, the U-Net model segmentation is better but loses some details in complex environments. However, the CBAM-HRNet based on CBAM with hrnetv2_w32 as the backbone network and Adam as the optimizer is better for segmenting wheat ear images. Moreover, it is less susceptible to other noise, can accurately segment the spike grains, and can be used to calculate the number of spike grains.

**Fig. 10:**
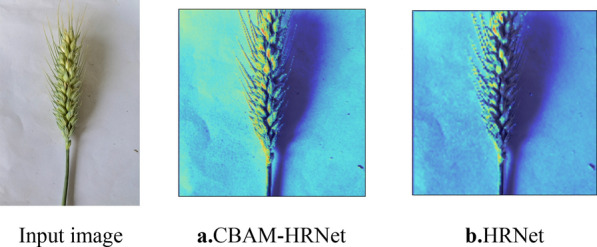
Grad-CAM visualization results highlight the important regions of the training model predicted. We compared the visualization results of CBAM-HRNet and HRNet. The Grad-CAM visualization was calculated on the final convolutional output. Correctly predicted categories are shown in red and incorrectly predicted in blue. CBAM supports the network in correcting the predictions and improving the effect of target segmentation

Gradient-weighted class activation mapping (Grad-CAM) was applied to CBAM-HRNet and HRNet using images from the wheat ears test set to highlight important regions and to make the role of CBAM in feature enhancement and performance improvement more apparent. Grad-CAM is a gradient-based visualization method that calculates the importance of spatial locations in a convolutional layer relative to a unique class [32]. We investigate how CBAM can help the network enhance discrimination by highlighting regions the network considers important for predicting classes. The visualization results of CBAM-HRNet were compared with those of HRNet. The visualization results are shown in Fig. 10.

As shown in Fig. 10, CBAM-HRNet outperforms HRNet in accurately segmenting the wheat grain. CBAM helps HRNet extract more relevant information from the wheat grain region and assists in aggregating its features. The CBAM feature refinement process enables the network to utilize the given features efficiently and rectify their predictions. Therefore, CBAM-HRNet is an efficient deep learning model for better segmentation of wheat grains [33].

### Accuracy analysis and evaluation of wheat grain counts

**Fig. 11:**
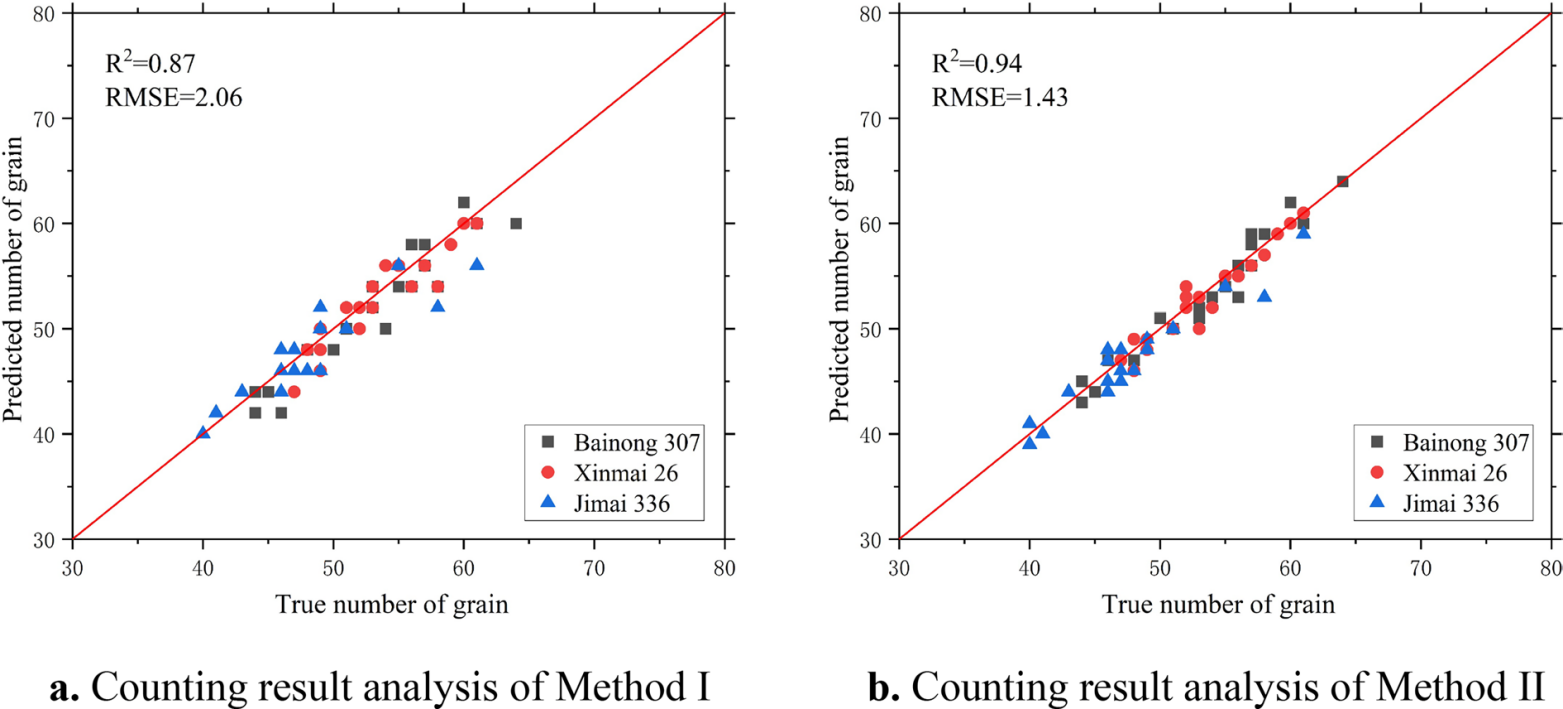
Analysis of counting results of the spike grain count model. Method I is two times the number of grains on one side of the ears; Method II is the sum of the grains on both sides

We selected 30 sample images of each wheat variety and segmented the spike grains, counted by the CBAM-HRNet based on CBAM and the image processing algorithm. Then, we calculated the number of individual wheat spikes grain using two methods obtained from the agronomic knowledge and compared results with manual counting (Fig. 11). The analysis of the counting results and the evaluation of the counting accuracy of wheat spike grains (Table 4) were conducted for the spike grain count model. From Fig. 11 and Table 4, Method I (two times the number of grains on one side of the spike as the total number of grains) was used to count the grains of the three varieties of wheat spike images with an RMSE of 1.89, an MAE of 1.53, an MRE of 2.91%, and an R^2^ of 0.85. Method II (the sum of the number of grains on both sides of the spike as the total number of grains) was used with an RMSE of 1.41, an MAE of 1.15, an MRE of 2.09%, and an R^2^ of 0.92. Our results show that the method is more accurate than the traditional image processing algorithm in counting wheat spike grains, with lower MAE and MRE and a better fit between the predicted and true values. The results obtained for the same wheat varieties using the same method under different nitrogen fertilizer treatments do not differ significantly, indicating that different nitrogen fertilizer treatments have a small effect on the counting results. Therefore, by applying this method to different varieties of wheat spikes, the accuracy of counting the number of spike grains can be greatly improved, and the automatic counting of spike grains of a single wheat plant with higher accuracy can be achieved [34].

**Table 4:** Evaluation of counting accuracy of wheat spike grain

Wheat varieties	Nitrogen fertilizer treatment	Counting accuracy of method I				Counting accuracy of method II			
		Mean absolute error	Mean relative error/%	RMSE	R^2^	Mean absolute error	Mean relative error/%	RMSE	R^2^
Bainong 307	N15	1.42	2.52	1.66	0.85	0.80	1.35	1.18	0.95
	N19	1.25	2.36	1.48	0.88	1.15	1.70	1.23	0.94
Xinmai 26	N15	1.75	3.25	2.16	0.84	1.20	2.17	1.26	0.92
	N19	1.20	2.29	1.55	0.87	1.10	2.16	1.32	0.93
Jimai 336	N15	1.80	3.59	2.19	0.81	1.40	2.88	1.73	0.89
	N19	1.75	3.47	2.31	0.82	1.23	2.26	1.74	0.91

The counting accuracy of Method II is higher than Method

### Repeatability across different varieties

The reproducibility and performance of the two counting methods were further assessed by using 50 images of five wheat varieties taken in May 2023 at the Yuanyang Science and Education Park of Henan Agricultural University. As before, manual counts were used as the validation data. The statistical summary results of the two counting methods are given in Table 5.

**Table 5:** Relationships between counting with methods I and II and manual counting of 5 varieties

Wheat varieties	Counting accuracy of method I				Counting accuracy of method II			
	Mean absolute error	Mean relative error/%	RMSE	R^2^	Mean absolute error	Mean relative error/%	RMSE	R^2^
Bainong 4199	2.90	2.78	3.27	0.83	2.10	2.56	2.51	0.90
Kexing 3302	1.60	2.41	2.14	0.85	1.40	2.32	1.67	0.91
Yangmai 15	3.60	3.04	4.56	0.81	1.90	2.56	2.02	0.92
Yunong 904	4.10	3.28	4.44	0.82	2.60	2.81	3.52	0.89
Zhengmai 136	1.60	2.37	2.14	0.86	1.90	2.47	2.88	0.87
All cultivars	3.74	4.23	3.74	0.81	1.98	2.75	2.60	0.91

**Fig.12:**
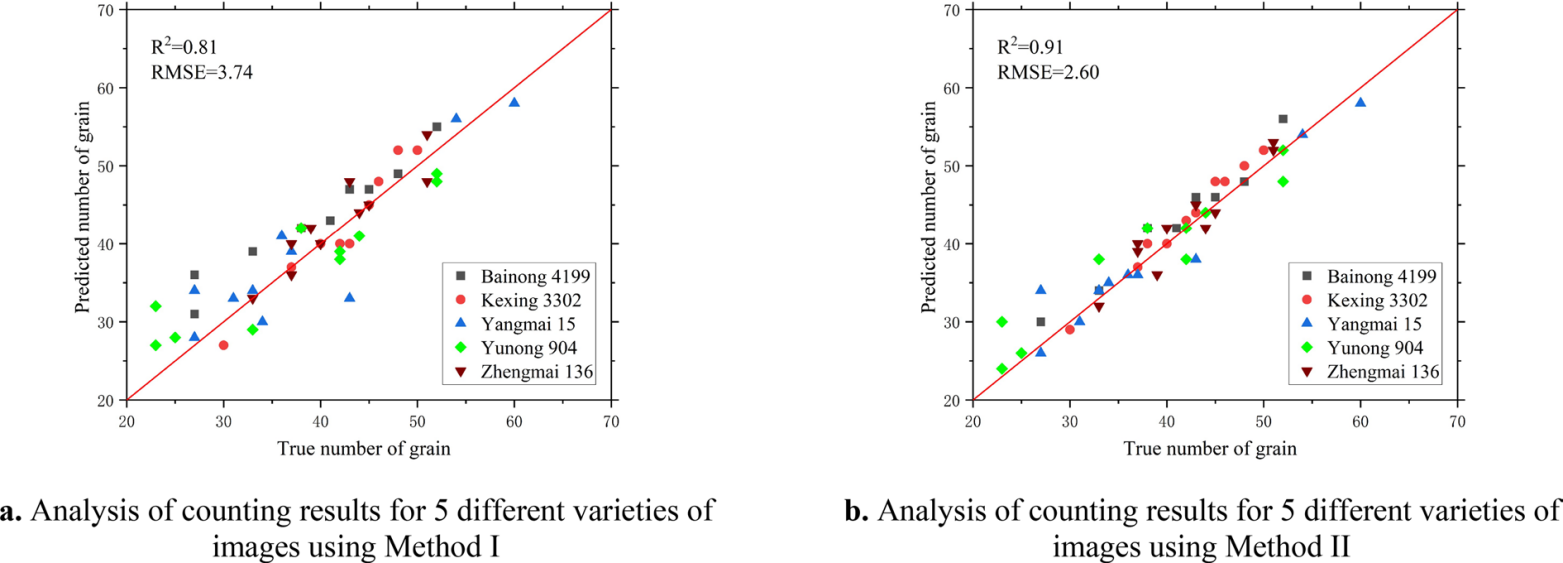
Analysis of counting results of for 5 different varieties of images using two different methods

The results show a high level of agreement between the 10 varieties (Fig. 12). While maintaining similar correlations, R^2^ decreased for both methods and the mean absolute error increased for both methods (Table 5). The R^2^ for these five varieties using both methods of counting was close to the previous three varieties, with Zhengmai 136 performing best (R^2^ = 0.86, RMSE = 2.14, MAE = 1.60, Table 5) and Yangmai 15 performing worst (R^2^ = 0.81, RMSE = 4.56, MAE = 3.60, Table 5) when counting using Method I; Yangmai 15 performed best (R^2^ = 0.92, RMSE = 2.02, MAE = 1.90, Table 5) and Zhengmai 136 performed worst (R^2^ = 0.87, RMSE = 2.88, MAE = 1.90, Table 5) when counted using Method II. This indicates that the genotypes of the different varieties had less influence on the count results. These results suggest that more genotype images are needed to facilitate model training and to improve the accuracy of the segmentation model for achieving higher counting accuracy.

## Discussion

The results showed that the CBAM-HRNet based on CBAM and image processing algorithm produced the wheat spike grain counts consistent with the manual counting results (Fig. 11 and Table 4). Alkhudaydi et al. [35] showed that the deep learning segmentation model demonstrated excellent performance during the filling stage, indicating that high-quality images could be captured at the late filling stage of wheat grains.

However, different varieties had a small effect on the segmentation and counting results (Fig. 12 and Table 5). Even though a small sample size of training data for the model was collected on May 15 and June 08 in 2022, the model still achieved good recognition results for images captured on other dates [5]. Capturing images using a mobile device parallel at a suitable height in a clear and cloudy environment is the most practical and convenient option, as it allows a clear view of the phenotypic wheat ear phenotypic details. Compared to CMOS industrial cameras [7] and UAVs for image acquisition, mobile devices are a more practical, convenient, and applicable option.

Zhao et al. [5] were the first to determine the correlation between the structural image features of rice spikelets and the number of grains. Du et al. [7] replicated this approach to wheat research and demonstrated the feasibility of indirectly obtaining the number of spikelet grains through the number of spikelet area pixels. Compared with the indirect method, the direct counting of wheat spike grains eliminates indirect errors and improves counting efficiency and accuracy. The spike grain count model uses the results predicted by the deep learning segmentation model as input and counts the spike grains directly through a series of image processing algorithms. This approach allows faster and more accurate counting of wheat grains than using a processed RGB image as input.

The proposed CBAM-HRNet based on CBAM outperformed other segmentation models such as HRNet, PSPNet, DeeplabV3+, and U-Net (mIoU = 85.21%, Table 2) in segmenting wheat spike grains during the filling period. With a slight increase in parameters and computation, CBAM-HRNet reduced the training time and could predict a wheat image at around 12.75 ms (Table 3), achieving better prediction results. The OpenCV image processing algorithm was also used to segment the predicted image for counting wheat spike grains. Compared with traditional image processing algorithms [12], this method dramatically improves the accuracy of the spike grains recognition and enables the automatic counting of the grains of a single wheat ear with higher accuracy [36].

This research has contributed to developing a low-cost, rapid, easy-to-implement system for counting wheat spike grains. Current research has mainly used measures such as fixing the shooting height [37] or placing references as ground standards [38], which reduces the method’s utility. Our future research will investigate using UAVs for low-altitude image acquisition for wheat spike grain counting.

## Conclusion

This study proposes a CBAM-HRNet to accurately count wheat spike grain numbers, incorporating CBAM combined and an image processing algorithm. The main conclusions are as follows:

1. A CBAM was added to the original HRNet to increase the efficiency of feature extraction and prevent the weights from being too random. The goal is to address the problems of complex semantic information of wheat ears and the severe phenomenon of sticking and covering between spike grains. After comparing various models, CBAM-HRNet took relatively less time to train under slightly more parameters, improving training efficiency and slightly increasing prediction time but achieving better prediction results. The CBAM-HRNet based on CBAM proved more robust than other network models in predicting better results.
2. Our study implemented the CBAM-HRNet to train, validate, and test images of the wheat ear dataset. We evaluated our segmentation accuracy using metrics like mIoU and achieved significant results. Our model outperformed other segmentation models, such as HRNet, PSPNet, DeeplabV3+, and U-Net, highlighting its superior generalization ability. Our segmentation accuracy of wheat spike grain of 92.04%, the mIoU value of 85.21%, the mPA value of 91.16%, and the recall of 91.16% demonstrate our model’s exceptional performance.
3. The use of an image processing algorithm to count the grains of wheat spikes was thoroughly investigated in this study. Two methods of calculating the total number of wheat spike grains are identified based on agronomic knowledge. By calculating the fit and error and comparing the manual count and the predicted value in the traditional image processing algorithm, Method I (the total number of grains is twice the number of grains on one side of the spike) and Method II (the sum of the number of grains on both sides of the spike is the total number of grains) can improve the recognition accuracy of the number of spike grains and realize the automatic counting of the grains of a single wheat spike with higher accuracy. Method I is more convenient because it only requires one side of the spike image to achieve high accuracy. Contrarily, Method II is more complex because it requires a complete image of both sides of the spike, but its spike count is more accurate than that of Method I. Nevertheless, the accuracy of both methods is virtually unaffected by different nitrogen fertilizer treatments.
4. Our model can be used to estimate the number of wheat spike grains and improve the efficiency of wheat yield estimation. This model can revolutionize wheat yield estimation and provide agricultural workers with a fast, automated, high-throughput counting system for the wheat spike grain. The method applies to the division and counting of wheat spike grains, which can be applied to the division and counting of other plants. Our future work is to reduce the cost of image acquisition and improve the counting accuracy and application of the method.

## Data Availability

The datasets used for the analysis are available from the corresponding author upon reasonable request.
